# Fruits as Food and Medicine

**Published:** 1885-02

**Authors:** 


### FRUITS AS FOOD AND MEDICINE.

Of all the fruits with which we are blessed, the peach is the most delicious and digestible. There is nothing more palatable wholesome and medicinal than good ripe peaches. They should be ripe, but not over ripe and half rotten; and of this kind they may make a part of either meal, or be eaten between meals; but it is better to make them part of the regular meals. It is a mistaken idea that no fruit should be eaten at breakfast. It would be far better if our people would eat less bacon and grease at breakfast and more fruit. In the morning there is an acrid state of the secretions, and nothing is so well calculated to correct this as cooling sub-acid fruits, such as peaches, apples, etc. Still, most of us have been taught that eating fruit before breakfast is highly dangerous. How the idea originated I do not know, but it is certainly a great error, contrary to both reason and facts.

The apple is one of the best of fruits. Baked or stewed apples will generally agree with the most delicate stomach and are an excellent medicine in many cases of sickness. Green or half-ripe apples stewed and sweetened are pleasant to the taste, cooling, nourishing and laxative, far superior, in many cases, to the abominable doses of salts and oil usually given in fever and other diseases. Raw apples and dried apples stewed are better for constipation than liver pills.

Oranges are very acceptable to most stomachs, having all the advantages of the acid alluded to; but the orange juice alone should be taken, rejecting the pulp.

The same may be said of lemons, pomegranates, and all that class. Lemonade is the best drink in fevers, and when thickened with sugar is better than syrup of squills and other nauseaus in many cases of cough.

Tomatoes act on the liver and bowels, and are much more pleasant and safe than blue mass and "liver regulators." The juice should be used alone rejecting the skins.

The small seeded fruits, such as blackberries, figs, raspberries, currants, and strawberries, may be classed among the best foods and medicines. The sugar in them is nutritious, the acid is cooling and purifying, and the seeds are laxative.

We would be much the gainers if we would look more to our orchards and gardens for our medicines, and less to our drug stores. To cure fever or act on the kidneys, no fabrifuge or diuretic is superior to watermelon, which may with very few exceptions, be taken in sickness and health in almost unlimited quantities, not only without injury, but with positive benefit. But in using them, the water, or juice, should be taken, excluding the pulp; and the melon should be fresh and ripe, but not over ripe and stale.

